# Challenges in Diagnosis and Treatment of a Cervical Spinal Cord Injury Patient with Melanoma, Adenocarcinoma, and Hepatic and Osteolytic Metastases: Need to Implement Strategies for Prevention and Early Detection of Cancer in Spinal Cord Injury Patients

**DOI:** 10.1155/2012/531214

**Published:** 2012-11-20

**Authors:** Subramanian Vaidyanathan, Paul Mansour, Peter L. Hughes, Fahed Selmi, Gurpreet Singh, Kamesh Pulya, Bakul M. Soni

**Affiliations:** ^1^Regional Spinal Injuries Centre, Southport and Formby District General Hospital, Town Lane, Southport PR8 6PN, UK; ^2^Department of Cellular Pathology, Southport and Formby District General Hospital, Town Lane, Southport PR8 6PN, UK; ^3^Department of Radiology, Southport and Formby District General Hospital, Town Lane, Southport PR8 6PN, UK; ^4^Department of Urology, Southport and Formby District General Hospital, Town Lane, Southport PR8 6PN, UK; ^5^Department of Medicine Cardiology Services, Southport and Formby District General Hospital, Town Lane, Southport, PR8 6PN, UK

## Abstract

A male tetraplegic patient with, who had been taking warfarin, developed haematuria. Ultrasound scan revealed no masses, stones, or hydronephrosis. Urinary bladder had normal configuration with no evidence of masses or organised haematoma. Urine cytology revealed no malignant cells. Four months later, CT urography revealed an irregular mass at the base of urinary bladder. Cystoscopic biopsy revealed moderately differentiated adenocarcinoma, which contained goblet cells and pools of mucin showing strongly positive immunostaining for prostatic acid hosphatase and patchy staining for prostate specific antigen. Computed Tomography revealed multiple hypodense hepatic lesions and several osteolytic areas in femoral heads and iliac bone. With a presumptive diagnosis of prostatic carcinoma, leuprorelin acetate 3.75 mg was prescribed. This patient expired a month later. *Conclusion*. (i) Spinal cord injury patient, who passed blood in urine while taking warfarin, requires *repeated* investigations to look for urinary tract neoplasm. (ii) Anti-androgen therapy should be prescribed for 2 weeks prior to administration of gonadorelin analogue to prevent tumour flare causing bone pain, bladder outlet obstruction, uraemia, and cardiovascular risk due to hypercoagulability associated with a rapid increase in tumour burden. (iii) Spinal cord physicians should adopt a caring and compassionate approach while managing tetraplegic patients with several co-morbidities, as aggressive diagnostic tests and therapeutic procedures may lead to deterioration in the quality of life.

## 1. Introduction

Spinal cord injury patients, who have long-term indwelling urinary catheter, may pass blood-stained urine as a result of catheter trauma or urine infection. If a tetraplegic patient who has been taking warfarin passes blood in urine, haematuria may be a manifestation of serious urological disease or a common result of anticoagulant therapy. It is advisable to repeat urologic investigations if the initial tests revealed no abnormality of kidneys and urinary bladder. We present a spinal cord injury patient, who had been prescribed warfarin for atrial fibrillation and developed haematuria. The aim of this paper is to illustrate the challenges we encountered in making a precise diagnosis and subsequent management. 

## 2. Presentation

A Caucasian male patient sustained tetraplegia at C-7 level (Frankel Grade C) in July 2001 when he was 72 years old. This patient was travelling in a boat; the boat split and he was thrown backwards. He developed pulmonary embolism in August 2001. A vena cava filter was inserted. He had atrial fibrillation also. This patient was prescribed digoxin and warfarin to be taken indefinitely. The target international normalised ratio was 3.0. He was managing his bladder by suprapubic cystostomy. 

In October 2010, this patient passed blood in urine. Warfarin was stopped and haematuria subsided. Subsequently, warfarin was restarted. Then haematuria recurred. This cycle of events happened three times. Warfarin was discontinued and this patient was prescribed enoxaparin sodium. But haematuria recurred. Then the dose of enoxaparin was reduced. Even then, this patient passed blood in urine. On the assumption that urine infections contributed to occurrence of haematuria, this patient was prescribed antibiotics. 

In April 2011, ultrasound examination of urinary tract was performed. Both kidneys were of normal site, size, and smooth outline. No masses, stones, or back pressure changes were seen. Urinary bladder had normal configuration with suprapubic cystostomy balloon seen in place. There was no evidence of masses or organised haematoma. Cytology of urine revealed very large numbers of neutrophil polymorphs, suggesting an acute urinary tract infection. Red blood cells are also present, together with only occasional epithelial cells. No malignant cells are identified.

**Figure 1:**
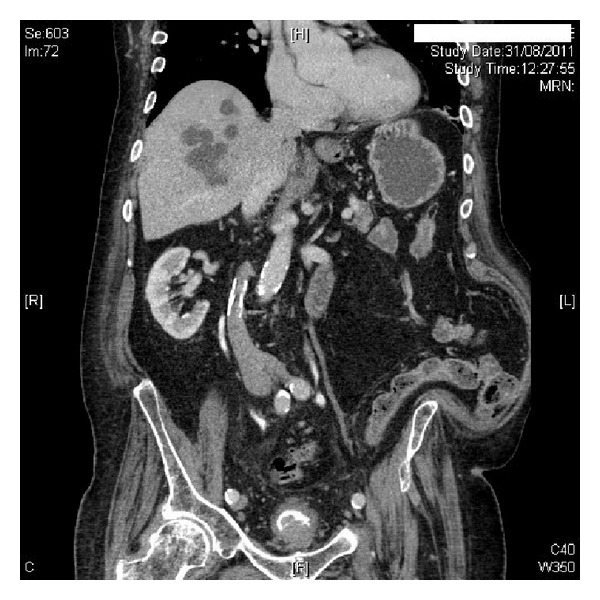
CT urography (coronal view) revealed an irregular mass at the base of urinary bladder.

**Figure 2:**
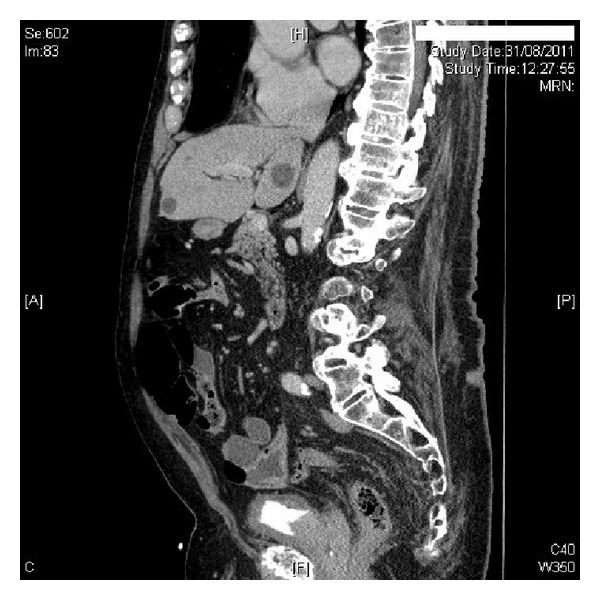
CT urography (sagittal view) revealed an irregular mass at the base of urinary bladder, which was likely to be an irregularly enlarged median lobe of prostate rather than intrinsic bladder neoplasm.

After four months, CT Urogram was performed, which revealed small left renal cyst. There was no space occupying lesion in the kidneys. Pelvicalyceal systems and ureters were normal. The urinary bladder was thick walled. There was an irregular mass at the bladder base, which was likely to be an irregularly enlarged median lobe of prostate rather than intrinsic bladder mass (Figures 1 and 2). This mass was very close to the ureteric orifices, which were at risk of involvement. Total prostate specific antigen level was 21.16 ug/L (reference range: 0.00–5.00).

In October 2011, this patient underwent erbium laser treatment of melanoma nodules on the right foot around the heel and the ankle under local anaesthetic. Subsequently, cystoscopy was performed through suprapubic cystostomy. Polypoid tumour was seen in the base of the bladder. Biopsy was taken. 

**Figure 3:**
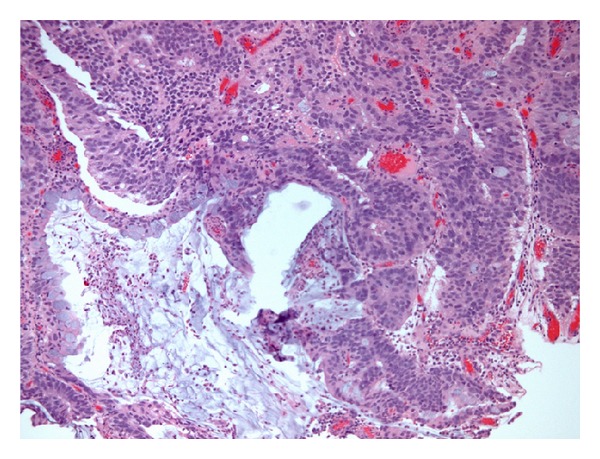
Medium-power photomicrograph of tumour showing abundant acid mucin (amorphous pale blue material within glands and extravasated from large gland at bottom left): confluent glands define a Gleason 4 pattern.

**Figure 4:**
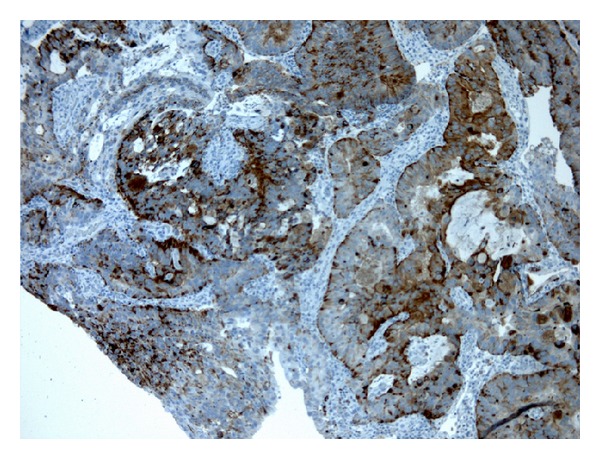
Medium-power image of immunostaining with anti-prostatic acid phosphatase, showing strong (brown) cytoplasmic staining.

Histology revealed high-grade adenocarcinoma, which in many areas contained goblet cells and pools of mucin (Figure 3). The surface urothelium was not apparent. Immunostaining showed strong positive staining for prostatic acid phosphatase (Figure 4), moderate patchy staining for prostate specific antigen; focal staining for cytokeratin 7 and Cytokeratin 20, and negative staining for 34*β*E12. 34*β*E12 reacts with the basal cells in benign prostatic acini, including basal cell hyperplasia and atypical adenomatous hyperplasia, as well as in prostatic intraepithelial neoplasia (PIN), but not in prostatic adenocarcinoma (in which, by definition, basal cells are absent); thus negative staining correlated with the diagnosis of prostatic adenocarcinoma. A rectal primary was unlikely because of largely negative staining for cytokeratin 20 and CDX2, a marker for gastrointestinal differentiation, especially colorectal.

Computed tomography of abdomen and pelvis was performed to assess extraprostatic extension of the tumour. There was no significant extravesical or extraprostatic extension. There was no evidence of pathologically enlarged regional lymph nodes. Multiple small osteolytic areas were seen involving most of the examined bony skeleton especially, at the femoral heads and iliac bone; multiple hepatic focal lesions were present, findings in keeping with metastasis.

We were unable to make a definite diagnosis of prostatic carcinoma because of the following reasons.

1. Prostatic adenocarcinoma is either acinar or ductal, and within the group of acinar tumours, there is a “colloid and signet ring variant.” However, the histological images did not favour a diagnosis of colloid and signet ring variant, as mucin was present only within the glands and not within the stroma.
2. Prostate specific antigen is specific for prostatic carcinoma whereas prostatic acid phosphatase is not [1]. In this patient, staining for prostate specific antigen was moderate and patchy.
3. Computed tomography revealed osteolytic areas involving the femoral heads and iliac bone, and multiple hepatic focal lesions, the appearance of which suggested metastasis. Such a pattern of metastatic spread was very unusual for prostatic cancer.

Although the morphological appearances favoured a likely diagnosis of primary prostatic adenocarcinoma, there were significant factors such as patchy staining for prostate specific antigen and pattern of metastases, which raised doubts as to whether this patient really had primary prostatic carcinoma. Biopsy of hepatic and bone metastases would have helped to reach a more precise diagnosis, but this patient was taking warfarin; he had pressure sore; he had atrial fibrillation. In view of the fact that the patient had two malignancies (melanoma and adenocarcinoma) and several comorbid conditions, we decided to adopt a conservative approach. Our aim was to avoid any intervention which might lead to further deterioration in the quality of life. With a presumptive diagnosis of prostate cancer, leuprorelin acetate 3.75 mg was prescribed. Anti-androgen treatment was not started before the gonadorelin analogue therapy. This patient's condition deteriorated and he expired four weeks later.

## 3. Discussion

If this patient indeed had prostate cancer stage D-2 as defined by the Jewett staging system, during the first 1–3 weeks of therapy with luteinizing hormone-releasing hormone agonist, there could be an initial increase in testosterone, a condition known as “flare.” Blockade of flare can be accomplished with a number of agents, including flutamide, bicalutamide, nilutamide, diethyl stilbestrol, ketoconazole, and cyproterone acetate. Evidence from the early use of luteinizing hormone-releasing hormone agonists suggested that flare could be serious in nature, with exacerbation of pain, increase in uraemia, development of neurologic sequelae, and possibly death [2]. The use of luteinizing hormone-releasing hormone analogues in patients with stage D-2 disease is associated with clinical flare in approximately 10% of patients. In addition to bone pain, spinal cord compression, and bladder outlet obstruction, another potentially severe side effect is cardiovascular risk arising from hypercoagulability associated with a rapid increase in tumour burden [3]. Thompson recommended that androgen blockade should be used in patients with advanced disease, as evidence suggested that with flare blockade, acute complications were extremely uncommon [2]. When blockade of flare was not used, two deaths were reported from one institution. 

We do not know whether this patient manifested tumour flare, as biochemical investigations were not performed subsequent to administration of leuprorelin and whether tumour flare contributed to his demise. Recently, we started prescribing gonadotrophin-releasing hormone antagonist, degarelix to treat advanced hormone-dependent prostate cancer. Degarelix does not induce a testosterone surge or tumour flare; therefore, antandrogen therapy is not required. 

Antoniewicz and associates [4] reviewed 238 patients who were referred to urology service in 2007–2009. Of 238 patients, 155 (65%) individuals received anticoagulant drugs. Haematuria was found predominantly in patients over 65 years; 71% of patients had concomitant diseases: hypertension, coronary heart disease, arrhythmia, and end-stage renal disease. Abnormalities of urinary tract were found in 45 patients (19%). The cost-effectiveness analysis revealed that the cost of detecting a single neoplasm was 3252 Euro. These researchers recommended redefinition of the occurrence of haematuria from the current concept of a manifestation of a serious urological disease to a common result of a long-term anticoagulant therapy.

Spinal cord injury patients may present with advanced cancer, as symptoms may be either minimal or mimicking other inflammatory conditions, which are quite common in this population. In our centre, we had misinterpreted squamous cell carcinoma of urinary bladder as vesical abscess [5]. Pannek found that more than 60% of the spinal cord injury patients with bladder cancer initially presented with muscle-infiltrating bladder cancer [6]. Tetraplegic subjects are at risk for developing cardio-pulmonary complications following anaesthesia and major surgery. If urological surgery is performed without anaesthesia, tetraplegic patients are at risk for developing autonomic dysreflexia [7]. 

A retrospective study of all spinal cord injury veterans receiving care at all the Department of Veterans Affairs Medical Centers in United States of America, who subsequently underwent proctectomy for rectal cancer during fiscal years 1993–2002 was carried out by Singh and associates [8]. Only patients with spinal cord injury due to trauma who met American Spinal Injury Association type A criteria (complete cord injury) were analyzed. Forty percent of spinal cord injury patients had major comorbidities at admission. Postoperative complications occurred in 80% of patients. The complication rate and length of stay for spinal cord injury patients undergoing proctectomy for rectal cancer were higher than those reported for otherwise comparable neutrally intact patients. Singh and associates concluded that spinal cord injury should be considered a risk factor for adverse outcomes in operations for rectal cancer as in other major surgery. 

As our patient had two malignancies (melanoma and adenocarcinoma) with hepatic and osteolytic metastases, we prescribed gonadorelin analogue on the assumption that the adenocarcinoma arose from prostate and decided to avoid chemotherapy or surgical procedures including biopsies.

Our patient sustained spinal cord injury at the age of 72 years. Ten years later, he developed haematuria and cancer of prostate was detected. Patel and associates [9] found a lower prevalence of prostate cancer among veterans with chronic spinal cord injury in comparison with age-matched veterans without spinal cord injury. However, the prostate cancer detected in the patients with spinal cord injury tended to be of a more advanced stage and grade. Scott and associates [10] postulated that the difference was likely a result of the decreased use of prostate cancer screening in this population. At present, prostate specific antigen levels are not checked during followup of male spinal cord injury patients in the North West Regional Spinal Injuries Centre, Southport, UK. 

Middleton and associates from the University of Sydney, Australia, observed that among the first-year survivors, overall 40-year survival rates were 47 and 62% for persons with tetraplegia and paraplegia, respectively [11]. As spinal cord injury patients live longer, they are at increased risk for developing cancer. *Therefore, spinal cord physicians should consider implementing strategies towards prevention and early detection of cancer during followup of these patients.*

Several behaviours identified may become targets of prevention strategies to increase longevity, including smoking cessation, stopping binge drinking, avoiding overreliance on psychotropic prescription medications, and promoting daily activities away from home [12]. Krause and associates [13] investigated the causes of death in patients who were ≤50 years at the time of traumatic spinal cord injury. In patients surviving ≥10 years, paraplegia was associated with a higher life expectancy compared with tetraplegia, 34 and 25 years (*P* = 0.008), respectively, and the leading causes of death were septicemia (*n* = 14), ischemic heart disease (*n* = 10), neoplasms (*n* = 9), cerebrovascular diseases (*n* = 5), and other forms of heart diseases (*n* = 5). Septicemia, influenza/pneumonia, and suicide were the leading causes of death in tetraplegics, whereas ischemic heart disease, neoplasms, and septicemia were the leading causes of death in paraplegia.

Spinal cord physicians should encourage paraplegic and tetraplegic subjects to participate in cancer screening programmes and provide necessary facilities in spinal units. Implementation of this strategy will require additional resources, for example, admission of a tetraplegic subject in a spinal unit for bowel preparation and sigmoidoscopy or colonoscopy. *Therefore, healthcare providers should allocate funding not only for early transfer to a spinal unit for rehabilitation but also for prompt admissions during subsequent years in order to facilitate spinal cord injury patients to undergo investigations for early detection of cancer. *Sadly, in the present setup, some spinal cord injury patients experience delay in diagnosis and treatment of cancer and consequently have poor survival from cancer [14]. 

## 4. Conclusion

The learning points from this case are as follows. (1) Spinal cord injury patient, who passes blood in urine while taking warfarin, requires *repeated* investigations to look for urinary tract neoplasm. (2) In a patient with stage D-2 prostate cancer, anti-androgen therapy should be prescribed for 2 weeks prior to administration of gonadorelin analogue to prevent tumour flare causing bone pain, bladder outlet obstruction, uraemia, and cardiovascular risk due to hypercoagulability associated with a rapid increase in tumour burden. (3) Spinal cord injury patients may be at risk for diagnosis of prostate cancer in advanced stage as a result of decreased use of prostate cancer screening in this population. (4) Spinal cord physicians should adopt a caring and compassionate approach while treating tetraplegic patients, who have developed cancer with metastases. (5) As life expectancy of spinal cord injury patients increases, these patients are at risk for developing cancer; therefore strategies towards prevention and early detection of cancer should be implemented during follow-up.

## References

[1] Graddis TJ, McMahan CJ, Tamman J, Page KJ, Trager JB (2011). Prostatic acid phosphatase expression in human tissues. *International Journal of Clinical and Experimental Pathology*.

[2] Thompson IM (2001). Flare associated with LHRH-agonist therapy. *Reviews in Urology*.

[3] Bubley GJ (2001). Is the flare phenomenon clinically significant?. *Urology*.

[4] Antoniewicz A, Zapała L, Poletajew S, Borówka A (2012). Macroscopic hematuria—a leading urological problem in patients on anticoagulant therapy: is the common diagnostic standard still advisable?. *International Scholarly Research Network Urololgy*.

[5] Vaidyanathan S, Mansour P, Ueno M (2002). Problems in early diagnosis of bladder cancer in a spinal cord injurypatient: report of a case of simultaneous production of granulocyte colonystimulating factor and parathyroid hormone-related protein by squamous cellcarcinoma of urinary bladder. *BMC Urology*.

[6] Pannek J (2002). Transitional cell carcinoma in patients with spinal cord injury: a high risk malignancy?. *Urology*.

[7] Vaidyanathan S, Soni B, Selmi F (2012). Are urological procedures in tetraplegic patients safely performed without anesthesia? A report of three cases. *Patient Safety in Surgery*.

[8] Singh RK, Dharmasena D, Virgo KS, Tyson SE, Grossmann EM, Johnson FE (2008). Proctectomy for rectal cancer in patients with prior spinal cord injury. *Surgical Oncology*.

[9] Patel N, Ngo K, Hastings J, Ketchum N, Sepahpanah F (2011). Prevalence of prostate cancer in patients with chronic spinal cord injury. *Physical Medicine and Rehabilitation*.

[10] Scott PA, Perkash I, Mode D, Wolfe VA, Terris MK (2004). Prostate cancer diagnosed in spinal cord-injured patients is more commonly advanced stage than in able-bodied patients. *Urology*.

[11] Middleton JW, Dayton A, Walsh J, Rutkowski SB, Leong G, Duong S Life expectancy after spinal cord injury: a 50-year study.

[12] Thietje R, Pouw MH, Schulz AP, Kienast B, Hirschfeld S (2011). Mortality in patients with traumatic spinal cord injury: descriptive analysis of 62 deceased subjects. *Journal of Spinal Cord Medicine*.

[13] Krause J, Saunders L (2010). Risk of mortality and life expectancy after spinal cord injury: the role of health behaviors and participation. *Topics in Spinal Cord Injury Rehabilitation*.

[14] Vaidyanathan S, Soni BM, Singh G, Hughes PL, Mansour P, Oo T (2011). Delay in diagnosis of cancer as a patient safety issue—a root cause analysis based on a representative case report. *Patient Safety in Surgery*.

